# A comparison of design algorithms for choosing the training population in genomic models

**DOI:** 10.3389/fgene.2024.1462855

**Published:** 2025-02-13

**Authors:** Alexandra Stadler, Werner G. Müller, Andreas Futschik

**Affiliations:** Institute of Applied Statistics, Johannes Kepler University, Linz, Austria

**Keywords:** GBLUP, genomic selection, optimal design, training population selection, design of experiments (DoE)

## Abstract

In contemporary breeding programs, typically genomic best linear unbiased prediction (gBLUP) models are employed to drive decisions on artificial selection. Experiments are performed to obtain responses on the units in the breeding program. Due to restrictions on the size of the experiment, an efficient experimental design must usually be found in order to optimize the training population. Classical exchange-type algorithms from optimal design theory can be employed for this purpose. This article suggests several variants for the gBLUP model and compares them to brute-force approaches from the genomics literature for various design criteria. Particular emphasis is placed on evaluating the computational runtime of algorithms along with their respective efficiencies over different sample sizes. We find that adapting classical algorithms from optimal design of experiments can help to decrease runtime, while maintaining efficiency.

## 1 Introduction

Plant breeding has been done by humans for centuries and has only grown in importance in recent history due to increasing global demand for food. Nowadays, it is possible to incorporate genetic information in breeding programs to improve decisions on artificial selection (Hickey et al., 2017). Researchers have investigated different statistical modeling methods for data analysis in this field. However, there are still a number of open questions w.r.t. model-oriented experimental design for phenotypic data collection in plant breeding programs. This problem is well-known in the field as optimization of the training set for model fit in genomic selection. Particularly, the search for optimum experimental designs under the genomic best linear unbiased prediction (gBLUP) model has not been studied extensively.

This article provides insights into the optimal design problem in the gBLUP model that is frequently applied in genomic selection. Section 2 is concerned with some preliminary information about the gBLUP model and a review of the generalized coefficient of determination, which is necessary for the introduction of the CDMin-criterion, an established criterion in breeding experiments. The optimal design problem is specified in Section 3 with emphasis on the particularities and differences to optimal design of experiments in classical linear models. Subsequently, different algorithms for the heuristic search of exact optimal designs are outlined in Section 4. A related discussion in a similar context is provided in Butler et al. (2021). The algorithms mentioned in Section 4 are applied to data and compared to algorithms provided in the TrainSel R package by Akdemir et al. (2021). The setup for this comparison is described in Section 5 and results are stated w.r.t. criterion values achieved as well as runtime in Section 6. A discussion on extensions of this work is given in Section 7. We provide conclusions in the final Section 8.

## 2 The gBLUP model

The gBLUP model is a special case of a linear mixed model with individual-specific random effects. Consider the following linear mixed model

y=Xβ+Zγ+ε, (1)

where y is a n×1 -vector of observed values, X is an n×p matrix, with corresponding p×1 -vector β of fixed effects, and $Z=I_{n}$ being the identity matrix of dimension n×n with corresponding n×1 -vector γ of individual-specific random effects. The n×1 -vector ε denotes the random errors. The vectors γ and ε are independent of each other and follow a normal distribution with

$$\left(\begin{array}{c} \gamma \\ \varepsilon \end{array}\right)∼N\left(\left(\begin{array}{c} 0\\ 0 \end{array}\right),\left(\begin{array}{cc} \sigma_{g}^{2}G & 0\\ 0 & \sigma_{e}^{2}I_{n} \end{array}\right)\right).$$

It is assumed that the genomic relationship matrix (GRM) G and the ratio $\lambda =\frac{\sigma_{e}^{2}}{\sigma_{g}^{2}}$ are known. Further, it is assumed that G is nonsingular. For convenience, define $V\;=\;\sigma_{g}^{2}ZGZ^{\prime }\;+\;\sigma_{e}^{2}I_{n}$ for subsequent formulas.

The GRM quantifies the degree of relatedness between plant lines in a breeding scheme. There are several ways to obtain such a matrix from genetic marker data (e.g., single-nucleotide polymorphism marker data). The calculation of the GRM is not the subject of this article, thus, for further information on this, the reader may want to refer to Clark and van der Werf (2013). For a detailed introduction to the gBLUP model and its relationship to other models in plant breeding, it is useful to refer to Habier et al. (2007) and VanRaden (2008).

The fixed and random effects in model (1) can be estimated/predicted via Henderson’s mixed model equations, see, e.g., Henderson (1975) and Robinson (1991). The estimators/predictors can be written as

$$\begin{array}{ll} \left(\begin{array}{c} \hat{\beta }\\ \hat{\gamma } \end{array}\right) & ={\left(D^{\prime }\sigma_{e}^{-2}I_{n}^{-1}D+\sigma_{g}^{-2}B\right)}^{-1}D^{\prime }\sigma_{e}^{-2}I_{n}^{-1}y\\ & ={\left(\sigma_{e}^{-2}D^{\prime }D+\sigma_{g}^{-2}B\right)}^{-1}\sigma_{e}^{-2}D^{\prime }y, \end{array}$$

with

$$D=\left(XZ\right),\;B=\left(\begin{array}{cc} 0 & 0\\ 0 & G^{-1} \end{array}\right).$$

The covariance matrix of the estimators/predictors is

$$\text{E}\left\{\left(\begin{array}{c} \hat{\beta }-\beta \\ \hat{\gamma }-\gamma \end{array}\right){\left(\begin{array}{c} \hat{\beta }-\beta \\ \hat{\gamma }-\gamma \end{array}\right)}^{\prime }\right\}={\left(\sigma_{e}^{-2}D^{\prime }D+\sigma_{g}^{-2}B\right)}^{-1}$$

$$\propto {\left(D^{\prime }D+\lambda B\right)}^{-1}=\left(\begin{array}{cc} H_{11} & H_{12}\\ H_{21} & H_{22} \end{array}\right),$$ (2)

where $H_{11}$ is of dimension p×p and it is the (proportional) covariance matrix of the fixed effects and correspondingly for the other submatrices.

The prediction variance of observed units is therefore

$$\text{Var}\left(\hat{y}\right)\propto \text{diag}\left\{D{\left(D^{\prime }D+\lambda B\right)}^{-1}D^{\prime }\right\}.$$

Let the covariance between random effects of units $x_{i}$ and $x_{j}$ be denoted by $\sigma_{g}^{2}G\left(x_{i},x_{j}\right)=\text{Cov}\left(\gamma_{i},\gamma_{j}\right)$ and $G\left(x_{i},x_{i}\right)=G\left(x_{i}\right)$ with the appropriate extension to a covariance matrix of several individuals.

The prediction variance of an unobserved individual is

$$\text{Var}\left({\hat{y}}_{0}\right)\propto d_{0}{\left(D^{\prime }D+\lambda B\right)}^{-1}d_{0}^{\prime }$$

where $d_{0}=\left(x_{0}\;z_{0}\right)$ is a row-vector of dimension n+p with $z_{0}=G\left(x_{0},X\right)G{\left(X\right)}^{-1}$ .

After model-fitting, breeders are mainly interested in the predicted random effects of all individuals potentially available for breeding. The predicted effects are called estimated genomic breeding values (GEBVs) and serve as a selection criterion, where the ranking of the breeding values is important (Jannink et al., 2010). The GEBVs of the individuals used for model fitting provide the predicted random effects $\hat{\gamma }$ . For individuals not included in the fitted model, the random effects must be rescaled to account for relationships between individuals. Let $z_{0}=G\left(x_{0},X\right)G{\left(X\right)}^{-1}$ , where $x_{0}$ denotes the vector of fixed effects for an unobserved individual. Then the GEBV of this individual is given by $z_{0}\hat{\gamma }$ (Clark and van der Werf, 2013).

Since breeders are mainly interested in the predictions of random effects, the goodness of a model shall be evaluated on the precision of the GEBVs in the sense that the ordering is most accurate. Arbitrary rescaling of the predicted random effects does not influence breeders’ selection decisions, hence, it is not important. To utilize a measure that is most useful in this respect, Laloë (1993) has introduced the generalized coefficient of determination with the purpose of quantifying the precision of the prediction.

Let the coefficient of determination (CD) of the random effect of unit i be defined by

$$CD\left(x_{i}|X\right)=\frac{\text{Var}\left({\hat{\gamma }}_{i}\right)}{\text{Var}\left(\gamma_{i}\right)}=1-\frac{\text{Var}\left(\gamma_{i}|{\hat{\gamma }}_{i}\right)}{\text{Var}\left(\gamma_{i}\right)},$$ (3)

where the design matrix in the model fit to predict $\hat{\gamma }$ is X .

As can be seen in Equation 3, the CD of an individual effect is a function of the variance ratio before and after the experiment. Consequently, CD is in the range of [0,1] . As described in Laloë (1993), the CD measures the squared correlation between the predicted and the realized random effect for an individual and thus measures the amount of information supplied by the data to obtain a prediction.

A matrix of CD values can easily be computed by

$$CD\left(X_{0}|X\right)=\text{diag}\left[\left\{G\left(X_{0},X\right)Z^{\prime }PZG\left(X,X_{0}\right)\right\}⊘G\left(X_{0},X_{0}\right)\right],$$

where ⊘ is the element-wise (Hadamard) division and $P\;=\;V^{-1}\;-\;V^{-1}X{\left(X^{\prime }V^{-1}X\right)}^{-1}X^{\prime }V^{-1}$ (Akdemir et al., 2021; Isidro y Sanchéz and Akdemir, 2021). Notice that X is the matrix of fixed effects for model fitting and $X_{0}$ is a design matrix of individuals. The concept of CD can be extended by introducing contrasts between individuals. For a given contrast c with appropriate dimension and |c|=0 , the respective CD values are given by

$$\text{diag}\left[\left\{c^{\prime }G\left(X_{0},X\right)Z^{\prime }PZG\left(X,X_{0}\right)c\right\}⊘c^{\prime }G\left(X_{0},X_{0}\right)c\right].$$

## 3 Optimal design problem

The design problem in the gBLUP model can be viewed as a selection of n experimental units out of N candidate units without replicates. Denote the design space as $X=\left\{x_{1},\ldots ,x_{N}\right\}$ , i.e., it is the set of all units that can potentially be included in the design. Note that the design matrix Z is not included in this definition, since it is the same regardless of the design for a given size n of the experiment, and replication of units is not considered. However, it is the case that the covariance of units $x_{i}$ in the model, with i=1,…,n , is different w.r.t. the design.

The exact design of size n<N is then written as $\xi_{n}$ and fulfills $\xi_{n}\subset X$ , $|\xi_{n}|=n$ . Define the remaining set as $\zeta =\left\{x_{i}\in X:x_{i}\notin \xi_{n}\right\}$ and notice that ξ∪ζ=X and ξ∩ζ=∅ .

Let the inverse of the (proportional) covariance matrix in Equation 2 be called the information matrix and denote it relative to a design $\xi_{n}$ by

$$M\left(\xi_{n}\right)=D^{\prime }\left(\xi_{n}\right)D\left(\xi_{n}\right)+\lambda B\left(\xi_{n}\right).$$

Due to the dependence of the GRM G on the exact design $\xi_{n}$ , the information matrix is not additive w.r.t. single experimental units. This makes the application of well-known experimental design theory on the classical linear model infeasible.

An optimal exact design $\xi_{n}^{*}$ of size n is typically defined as

$$\xi_{n}^{*}=\underset{\xi_{n}}{argmax}\Phi \left\{M\left(\xi_{n}\right)\right\},$$

for some optimal design criterion given by a function $\Phi :R^{q\times q}\mapsto R$ on the information matrix, where q=n+p .

Since breeders are mainly interested in the prediction of random effects in the gBLUP model, we restrict ourselves to criteria on the part of the covariance matrix that corresponds to random effects, i.e., to $H_{22}$ . In accordance with the literature on optimal design of experiments (cf. Atkinson et al., 2007), let the D-criterion on the covariance of random effects be defined by

$$\Phi_{1}\left\{M\left(\xi_{n}\right)\right\}=-\text{ln}|H_{22}\left(\xi_{n}\right)|.$$ (4)

The D-criterion is a standard criterion, which minimizes the generalized variance of the parameter estimates (i.e., predictions in this application). Hence, it measures the precision of the predictors. For a geometrical interpretation of the D-criterion, see, e.g., Atkinson et al. (2007), Fedorov and Leonov (2013).

Additionally, consider the so-called CDMin-criterion, defined as

$$\Phi_{2}\left\{M\left(\xi_{n}\right)\right\}=\min\left\{CD\left(X|\xi_{n}\right)\right\}$$

$$\Phi_{2}\left\{M\left(\xi_{n}\right)\right\}=\min\left\{CD\left(X|\xi_{n}\right)\right\}=\min\left(\text{diag}\left[\left\{G\left(X,\xi_{n}\right)Z^{\prime }PZG^{\prime }\left(X,\xi_{n}\right)\right\}⊘G\left(X\right)\right]\right).$$ (5)

The CDMin criterion is the default criterion in the R package TrainSel (see Akdemir et al., 2021). As previously mentioned in Section 2, the CD of an individual measures the amount of information supplied by an experiment to predict the random effect of an individual. The CDMin-criterion maximizes the minimum CD value over all individuals. Hence, it is driven by the intuition of controlling for the worst case. The generalization of the CD was initially proposed by Laloë (1993) and has since gained popularity in the field, see e.g., Rincent et al. (2012). There are other criteria related to the CD, e.g., the CDMean criterion, which instead optimizes for the average CD. Refer to Akdemir et al. (2021) for a discussion on the CDMin-criterion versus other criteria. The literature on optimization criteria for this task is very rich and current, see e.g., also Fernández-González et al. (2023), where the authors compare different criteria w.r.t. accuracy of the predicted traits. However, the main focus of this manuscript is not on the selection of a suitable criterion, but rather on the efficient optimization once a criterion has been chosen.

In the following, we will concentrate on the two criteria stated above for clarity, but our methodology and discussion may extend well beyond those.

## 4 Heuristic search for an exact optimal design

Experimental design optimization in the gBLUP model is challenging in the sense that the covariance matrix of the random effects is directly dependent on the design. In particular, the information matrix is not a weighted sum of unit-specific matrices, which does not allow for standard convex design theory from the classical linear model to be applied directly. Due to this fact, it seems most reasonable to consider only optimization methods for exact designs rather than approximate designs (cf. Prus and Filová, 2024).

Even though the information matrix in the gBLUP model is quite different from the standard case, it may be sensible to adopt some ideas on algorithms from the classical linear model. This section reviews and alters algorithms for the application at hand. Furthermore, a comparison to the algorithm and R package TrainSel by Akdemir et al. (2021) will be made in the subsequent section.

The optimization strategy proposed by Akdemir et al. (2021) (implemented in TrainSel) combines a genetic algorithm with simulated annealing to identify local maxima in a given design region. It does so in a brute-force way without relying on theoretical considerations w.r.t. the design of experiments. The TrainSel algorithm provides flexibility by allowing for user-specified criteria, which comes at the cost of perhaps more efficient optimization strategies for specific criteria. The implemented function TrainSel offers several parameters that can be modified for a given situation; most relevant to this article are the maximum number of iterations for the algorithm, as well as the number of iterations without (significant) improvement until the algorithm is considered to have converged.

The published article Akdemir et al. (2021) refers to the author’s publicly available R package on Github. Since then, the authors have made a major update to the repository in May 2024 (see Javier, 2024). The full functionality of the package is now under license, with the possibility of obtaining a free license for public bodies. A substantial part of the computations in this paper have been performed on TrainSel v2.0, the previously publicly available version on Github. To the best of our knowledge, this version is no longer available for download. We have made comparisons with the licensed version Trainsel v3.0, and have observed some changes, which will be discussed in subsequent sections. Most notably, the newest version permits the manipulation of even more hyperparameters in the optimization algorithms and provides more user-friendliness in the specification of these parameters by introducing settings related to the size and complexity of the optimization problem. The optimization algorithm itself seems to have improved as well.

In contrast to this approach, the literature on experimental design typically proposes exchange-type algorithms in the search for exact designs (cf. Atkinson et al., 2007). Exchange-type algorithms start with an initial design $\xi_{n}$ of size n (e.g., randomly sampled) and iteratively improve the design by exchanging units from the support and candidate set. In this case, the support and candidate set are mutually exclusive, and their union is the whole design space X . The units are exchanged either according to the best improvement in the criterion (Fedorov exchange, see Fedorov, 1972) or according to a greedy strategy (modified Fedorov exchange, see Cook and Nachtsheim, 1980). In the classical Fedorov exchange algorithm, the criterion value for all possible exchanges between the candidate and the support set is calculated at each iteration. This guarantees that the most beneficial exchange is made in each step, which leads to quick ascension. The disadvantage is that the number of computations in each iteration can be large depending on the size of the support and candidate set. In the modified Fedorov exchange, the exchange is performed as soon as an improvement is identified, hence, the ordering of the support and candidate set has an impact on the search. The advantage of the modified Fedorov exchange over the classical one is the reduced computational effort in each iteration if a beneficial exchange can be found quickly. The final exact designs are dependent on the initial design, i.e., for randomly sampled starting designs, the final designs are also subject to randomness.

Exchange algorithms have been applied in the field of plant breeding, e.g., in Rincent et al. (2012) and Berro et al. (2019). However, these articles have not included any guidance on selecting potential instances for exchange. Rather, they have randomly performed an exchange and recalculated the criterion value. If an improvement could be observed, the exchange was kept. We believe that exchange algorithms can be improved in two ways, either the known best exchange can be made at each step, or the chance of making a good exchange can be increased by ordering the support and candidate set by some heuristic. These two approaches will be discussed below.

Unfortunately, none of the algorithms can guarantee convergence to the global optimum. Thus, it is reasonable to restart the algorithms at different random seeds to obtain multiple solutions, as is typically advised in the literature (see, e.g., Atkinson et al., 2007). The design with the highest criterion value over all random restarts should ultimately be chosen.

In the classical linear model, the most beneficial exchanges under the D-criterion are performed by exchanging the unit with minimum prediction variance in the support set with the unit with maximum prediction variance from the candidate set (cf. Atkinson et al., 2007). The reasoning is not as clear-cut in the gBLUP model as will be argued below. A modified Fedorov exchange can be performed with different orderings of the candidate and support set, which may lead to different resulting designs as well as different runtimes.

As stated, the candidate set in the modified Fedorov exchange is usually ordered w.r.t. decreasing prediction variance for the D-criterion, i.e., candidates with a high prediction variance are considered first for inclusion in the support set. Since the gBLUP model relies on individual-specific random effects, the removal of a unit from the support set has non-trivial consequences on the variance of other units. This makes it relatively difficult to specify a sensible ordering of the support set for exchanges. Since the D-criterion minimizes the generalized variance of the predicted random effects in this application, it seems reasonable to remove units (and thereby also random effects in the model) that have a high prediction variance. As this is completely converse to the reasoning in the classical linear (or mixed effect) model, we will here vary the ordering of the support set to increasing, decreasing, and random for the subsequent examples.

For the CDMin-criterion, following a similar thought process, it seems reasonable to add units with a small CD from the candidate set in the modified Fedorov exchange. In turn, units with a high CD could be sensible candidates for removal from the support set. In an effort to compare the ordering strategies, the ordering of the support set is also performed in increasing, decreasing, and random order as for the D-criterion in our examples.

The Fedorov exchange algorithm has a natural ending, i.e., when no further improvement can be found for any exchange of a unit from the candidate set with a unit from the support set, the best solution obtained is returned. For the modified Fedorov exchange, the same stopping rule is given, but the path and solution obtained are not necessarily the same. On the other hand the TrainSel algorithm does not have such a natural ending. Instead, the user must specify a maximum number of iterations for the algorithm and a number of iterations without a (significant) improvement on the criterion value, which will terminate the algorithm. The threshold for minimum improvement must also be specified by the user. Termination of this algorithm is defined as early stopping when no significant improvement on the criterion value was attained for a specified number of iterations.

## 5 Comparison of different algorithms

In the previous section, three algorithms have been introduced: TrainSel, Fedorov exchange and modified Fedorov exchange, where in the modified Fedorov exchange the support set can be ordered in different ways.

A comparison between these algorithms is performed on a dataset of N=200 wheat lines given in the TrainSel R package (see Akdemir et al., 2021). The data consists of genotypes at 4,670 markers or as a precalculated relationship matrix of the 200 available individuals. Akdemir et al. (2021) name https://triticeaetoolbox.org as the original source of the data, which has undergone some additional preprocessing by the authors. We proceed with the GRM G over the 200 individuals as it is provided since the calculation is not the subject of this article. In this simulated design problem, we set the ratio of variances λ=1 and $R=I_{n}$ for a given sample size n . We further include an intercept in the model, i.e., X=1 .

The comparison is performed over ten restarts with different random seeds over the D-criterion in Equation 4 and the CDMin-criterion in Equation 5. Overall, the algorithms are run at sample sizes of n∈{10,20,…,80} . We eventually compare five different search strategies w.r.t. the criterion value obtained and the runtime of the algorithms.

Since there is no natural convergence of TrainSel, some arbitrary parameters must be selected for comparison to the other algorithms. There are conflicting goals in comparing criterion values and runtimes between algorithms, so the parameters were chosen such that TrainSel was not run unnecessarily long, but would converge to an appropriate solution. In particular, the maximum number of iterations was chosen to be 30⋅n , and the number of iterations without (sufficiently large) improvement before convergence was set to 100. All other parameters were set to the default values.

The results in the subsequent section refer to Trainsel v2.0, which is the algorithm described in the article Akdemir et al. (2021). Since the corresponding R package has since undergone major updates to version 3.0, we reproduce the plots in this section for the newer version as well. The set of hyperparameters in the newest version has increased in comparison to v2.0. First, we have strived to set parameters as similarly to version v2.0 as possible. Second, we have reproduced the design problems with the suggested hyperparameters in TrainSel v3.0 for large high-complexity designs. Only the maximum number of iterations and the number of iterations without improvement before convergence have been adjusted as in the computations with TrainSel v2.0. Since results were better in the second configuration, we only discuss this case in Section 6.1.

## 6 Results

**FIGURE 1:**
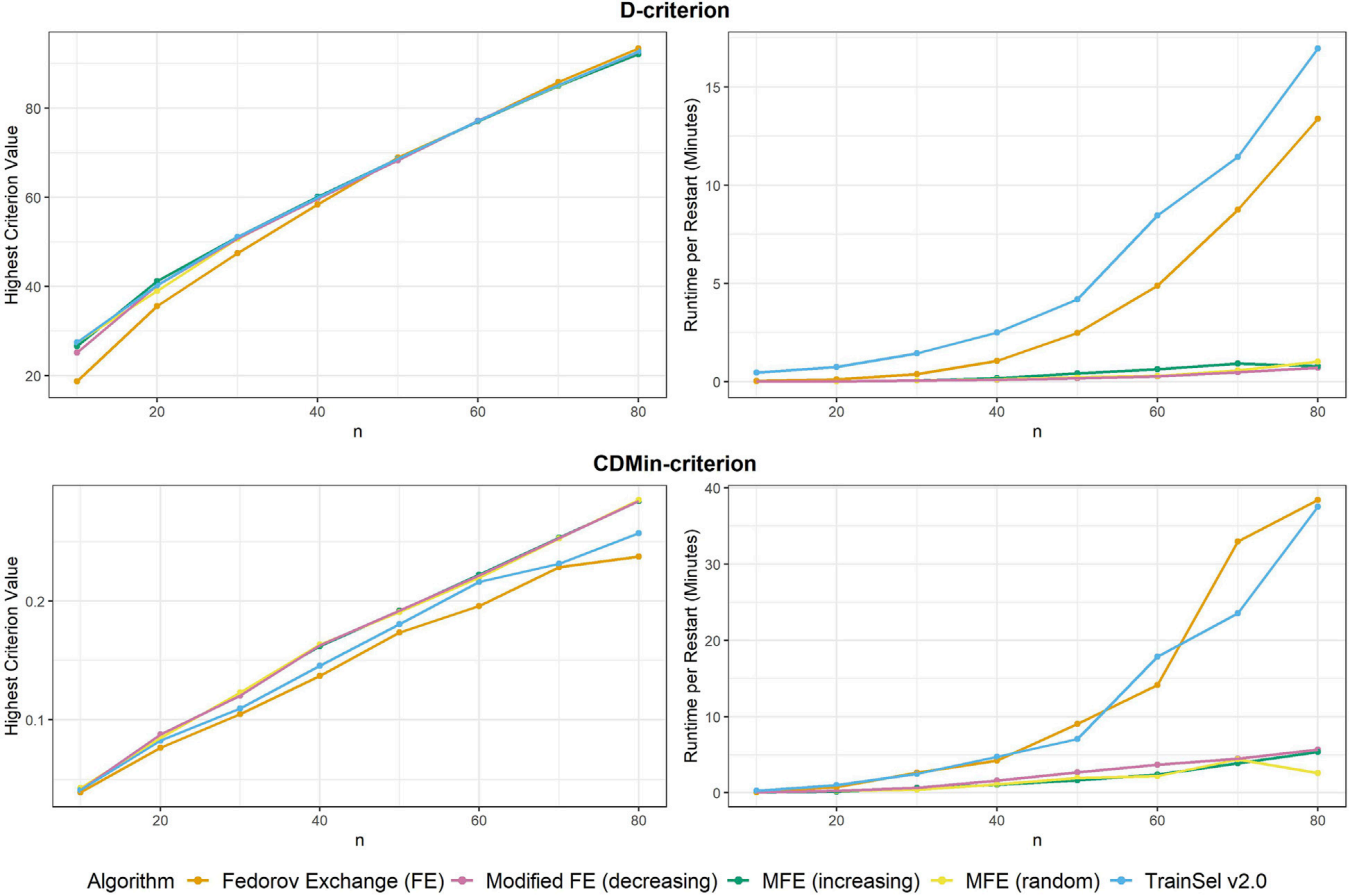
Highest criterion value and runtime per restart of different algorithms.

Firstly, the criterion values of different algorithms are compared to one another, secondly, runtimes are investigated and lastly, some convergence plots are examined. We start with comparisons for the D-criterion. The final criterion values and runtime per random restart can be seen in Figure 1. It is most notable that the Fedorov exchange algorithm does not perform well in terms of criterion value in small sample sizes, while it does obtain a slightly larger criterion value at n=80 than the other algorithms. In general, for sample sizes n≥30 , the criterion values for the TrainSel and (modified) Fedorov exchange algorithms are similar, while the runtime for the TrainSel algorithm as well as the Fedorov exchange exhibits rapid growth over increasing sample size as opposed to the modified Fedorov exchange.

Convergence was attained for all algorithms at all random restarts and sample sizes.

**FIGURE 2:**
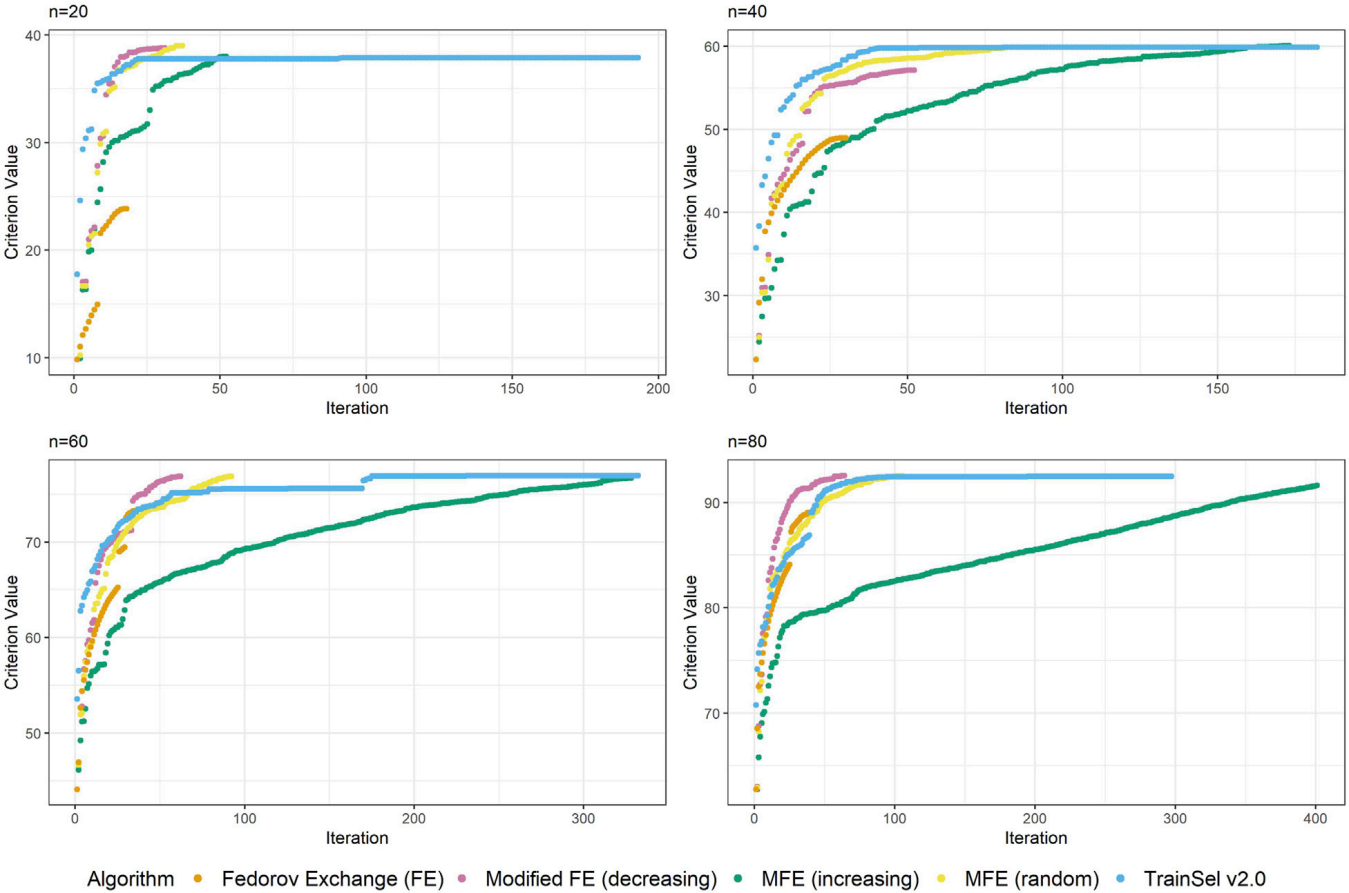
Convergence of algorithms over different sample size at one random seed (D-criterion).

Although the convergence plots of different algorithms do not directly imply anything related to runtime, it is still interesting to compare the ascension to criterion values, especially w.r.t. the different orderings of the support set in the modified Fedorov exchange algorithm. Therefore, Figure 2 displays criterion values per iteration for the different algorithms.

Figure 2 depicts the criterion values over iterations for sample sizes of n ∈ {20,40,60,80} at one random seed. The reader should be made aware that these graphs can look different at other random seeds, in particular for small sample sizes, but we deem this plot representative for showing some general properties. For a more comprehensive understanding, some more convergence plots at all random seeds are provided in the accompanying Supplementary material for this article.

One thing that is particular about the graphs is that the modified Fedorov exchange with ordering according to increasing variance (as is traditionally used in the classical linear model) displays a very slow ascension of the criterion value. Ultimately, the resulting criterion value at convergence is similar to the other algorithms, but if the algorithm were to be stopped early, even just a random ordering of the support set might result in higher criterion values. This may seem counterintuitive, but as has been mentioned in Section 5, by removing a unit from the support set, the whole individual-specific effect of this individual disappears from the model, hence units with high variance will be interesting candidates for removal. Obviously, there is a trade-off w.r.t. relationships (i.e., covariances) to other individuals, thereby making this reasoning non-trivial.

Another interesting fact is that at this random seed, TrainSel ascends rather quickly, but a plateau on the criterion value is retained for several iterations before convergence. This is due to the specified settings. Other settings would have resulted in different behavior (e.g., earlier stopping). It is not straightforward to choose settings *a priori* that will result in reasonable convergence behavior.

Now moving on to the CDMin-criterion, similar plots of criterion values and runtime are provided in Figure 1.

The results in the CDMin-criterion are different compared to the D-criterion in a number of ways. Firstly, it is very clear from Figure 1 that the Fedorov exchange is not well-suited for this problem, since it results in criterion values below the ones of all other algorithms and it has bad runtime properties. The same applies to the TrainSel algorithm, although it is still better than Fedorov exchange w.r.t. criterion values. One may observe that the modified Fedorov exchange yields similar criterion values regardless of the ordering of the support set, but a decreasing ordering of the support set w.r.t. CD results in a longer runtime. In general, the modified Fedorov exchange requires a longer runtime for the CDMin-criterion than for the D-criterion.

Convergence occurred for all algorithms at all random seeds and sample sizes, except for TrainSel, where 3 out of 10 random restarts did not converge for a sample size of n=10 , otherwise, convergence was always attained.

**FIGURE 3:**
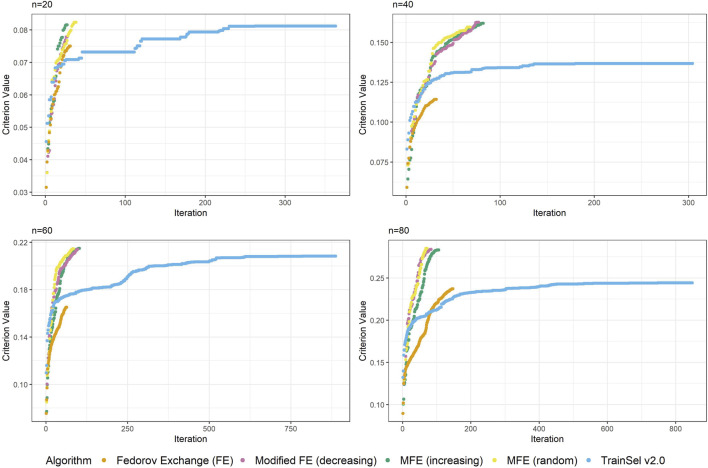
Convergence of algorithms over different sample size at one random seed (CDMin-criterion).

Again, Figure 3 looks quite different from Figure 2, i.e, the case of the D-criterion. In particular, the TrainSel algorithm does not ascend as quickly and there are still large jumps in the criterion value after a considerable number of iterations. It can also be seen from the second graph in this figure that TrainSel can be stuck in a local minimum that is not optimal and converges too early. Interestingly, the modified Fedorov exchange with the random ordering of the support set seems to have good ascension properties. As for the D-criterion, refer to the Supplementary Material to find convergence graphs for all random seeds at different sample sizes.

### 6.1 Comparison to TrainSel v3.0

We have observed improvements of the optimization algorithm in TrainSel in the last major update. Particularly, the optimization over the D-criterion is considerably improved. We reproduce Figures 1–3 subsequently to showcase the improvements.

We have included further computations for samples sizes n∈{90,100} for all algorithms except for the classical Fedorov exchange since this algorithm is not competitive with the other algorithms for large sample sizes.

**FIGURE 4:**
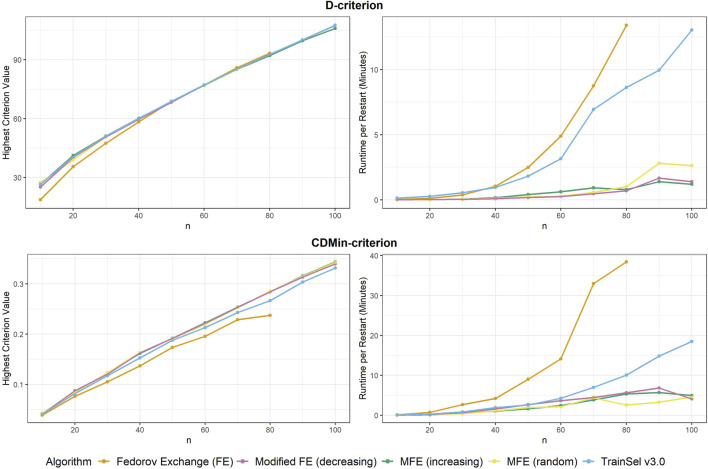
Highest criterion value and runtime per restart of different algorithms including TrainSel v3.0

**FIGURE 5:**
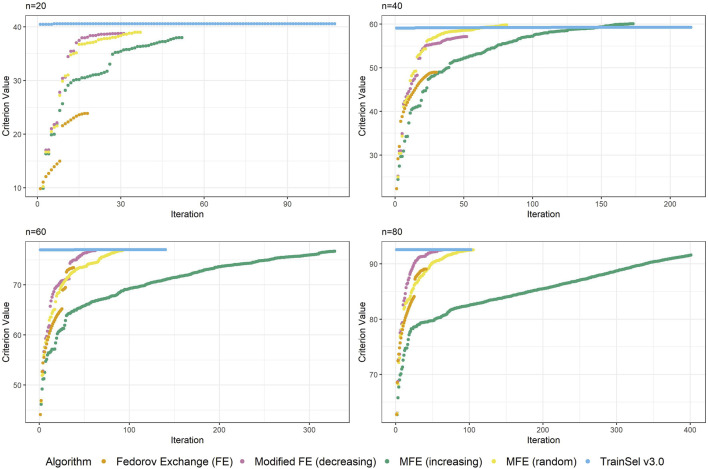
Convergence of algorithms over different sample size at one random seed (D-criterion) including TrainSel v3.0

From Figure 4 it seems that the runtime has improved substantially, while the criterion value is still equally high as in TrainSel v2.0 for the D-criterion. Looking at Figure 5, it is clear that TrainSel v3.0’s best solution is attained very quickly and the subsequent iterations are not necessary. While it is typically not possible to know this before optimization of a design problem, we can see *a posteriori* that the number of iterations until termination of the algorithm could have been chosen much smaller, which would improve runtime even further.

Seemingly, TrainSel v3.0 performs better than the previous version on the CDMin-criterion as well, both in runtime and criterion value attained. Nonetheless, even with these improved properties, the criterion values for larger sample sizes are still short of the ones attained with the modified Fedorov exchange algorithms. Also, the runtime still increases rapidly with the sample size, i.e., complexity of the design problem.

**FIGURE 6:**
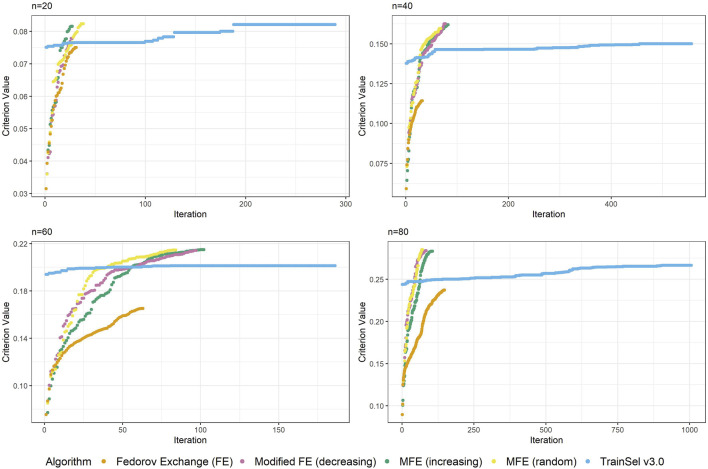
Convergence of algorithms over different sample size at one random seed (CDMin-criterion) including TrainSel v3.0

Comparing Figures 3, 6, one can conclude that the ascension to the criterion value is quicker in the beginning of the optimization in TrainSel v3.0, but the problem of getting stuck in a local optimum seems to remain. As emphasized previously, the tuning parameters could be adjusted to increase computational efforts put into the optimization, but it seems unclear how much improvement this can provide in general.

The reason for the performance improvement in TrainSel is difficult to explain. As the latest version of it is under license, it is not possible to directly access the base code to compare the changes that have been made to the optimization algorithm. The package is hosted in a GitHub project, which means that some changes to the top level documents of this package are visible between version 2.0 and 3.0. For example, the new version of TrainSel includes new dependencies on the R packages foreach and doParallel. As the names suggest, these packages are used for parallel computing. Even though the package parallel was previously used by TrainSel v2.0, some changes in parallel computing could have led to increased efficiency in the latest version. It is also possible that refinement to the optimization algorithm itself have led to this increased performance.

## 7 Discussion

This manuscript has showcased the application of different algorithms to a design problem in a single-trait gBLUP model in one environment. Naturally, in applications in plant breeding there may be interest in extending the model in a number of ways. For example, plant breeders are frequently intersted in performing experiments in multiple environments. This extension can be taken into account in the model, e.g., by including additional variables in the fixed effects term Xβ in the model of Equation 1. Additionally, there is special interest in modeling the interaction of genomic effects and environmental effects (G x E effects). This is important information for breeders, since it helps to evaluate the quality of particular plant lines in different environments. E.g., some may be very good in one particular environment, but perform badly when exposed to a “stressful” environment, like one with prolonged droughts.

There is a large interest in these effects, a motivational article on modeling G x E effects is e.g., van Eeuwijk et al. (2016). Again, the model can be extended to include this interaction term, which would correspond to another random effect in the model of Equation 1. The additional effect could be denoted $Z_{2}\gamma_{2}$ , where $Z_{2}$ is the matrix of interactions and $\gamma_{2}$ is the vector of random interaction effects. The random vector shall follow a normal distribution with mean 0 and some appropriate covariance matrix, e.g., $\Sigma_{\text{GxE}}=\Sigma_{\text{E}}⊗G$ , where $\Sigma_{\text{E}}$ could be a diagonal matrix with variances corresponding to the different environments and ⊗ is the Kronecker product.

Another possible extension could be the joint modeling of multiple traits that are of interest, either in one or multiple environments. For an exemplary article with stipulation and application of such a model to simulated and real data, see e.g., Montesinos-López et al. (2016).

The present manuscript was written such that the particularities of experimental design could most easily be understood. Therefore, extensions of this kind were omitted and focus was centered on the algorithmic optimization in such problems.

## 8 Conclusion

This article reviewed the optimal design problem in the gBLUP model and the main differences to the classical linear model. Some strategies for the search of exact optimal designs were discussed and put to use in a fictitious optimal experimental design problem with data from the TrainSel R package. A comparison w.r.t. criterion values and runtime was performed on the D- and CDMin-criteria over several sample sizes.

The results suggest that while TrainSel provides a lot of flexibility for users to optimize various design problems, it might be more efficient to rely on theoretical considerations and more classical algorithms in at least some applications on the gBLUP model. In particular, for the D-criterion, all algorithms resulted in similarly efficient designs for a sample size of n≥40 , but a much larger runtime was necessary for TrainSel 2.0. The newest licensed version of TrainSel has improved on runtime properties and optimization results. In general, for increasing sample size, TrainSel’s runtime grows much quicker than the ones for the modified Fedorov exchange.

It is quite interesting to note that the ordering of the support set in the modified Fedorov exchange according to decreasing variance seems to be beneficial and leads to quick ascension, as opposed to the traditional ordering by increasing variance.

For the CDMin-criterion (the default criterion in TrainSel), the Fedorov exchange is not suitable, since it provides smaller criterion values and has bad runtime properties. The TrainSel algorithm results in smaller criterion values as well and runtime increases rapidly and is much larger than for the modified Fedorov exchange. In the modified Fedorov exchange, it seems that all sortings of the support set provide similarly efficient designs, but sorting by decreasing CD still takes slightly longer. It is interesting to see that even random sorting of the support set for exchange seems to work quite well.

Overall it was demonstrated that classical exchange-type algorithms can typically obtain similarly efficient designs as TrainSel while substantially saving computational runtime. We expect this advantage to scale with respect to the increasing complexity of the respective problem.

## Data Availability

Publicly available datasets were analyzed in this study. This data can be found here: The data underlying this article are available in the R package TrainSel (see Akdemir et al., 2021). The authors name https://triticeaetoolbox.org as the original source of the data, which has undergone some additional preprocessing.
